# Development of an earthworm-based soft robot for colon sampling

**DOI:** 10.3389/frobt.2024.1309220

**Published:** 2024-02-07

**Authors:** Gongxin Li, Wei Qiu, Mindong Wang, Yazhou Zhu, Fei Liu

**Affiliations:** Key Laboratory of Advanced Process Control for Light Industry (Ministry of Education), Institute of Automation, Jiangnan University, Wuxi, Jiangsu, China

**Keywords:** soft robot, earthworm-like, soft sampler, colorectal cancer, colon sampling

## Abstract

Colorectal cancer as a major disease that poses a serious threat to human health continues to rise in incidence. And the timely colon examinations are crucial for the prevention, diagnosis, and treatment of this disease. Clinically, gastroscopy is used as a universal means of examination, prevention and diagnosis of this disease, but this detection method is not patient-friendly and can easily cause damage to the intestinal mucosa. Soft robots as an emerging technology offer a promising approach to examining, diagnosing, and treating intestinal diseases due to their high flexibility and patient-friendly interaction. However, existing research on intestinal soft robots mainly focuses on controlled movement and observation within the colon or colon-like environments, lacking additional functionalities such as sample collection from the intestine. Here, we designed and developed an earthworm-like soft robot specifically for colon sampling. It consists of a robot body with an earthworm-like structure for movement in the narrow and soft pipe-environments, and a sampling part with a flexible arm structure resembling an elephant trunk for bidirectional bending sampling. This soft robot is capable of flexible movement and sample collection within an colon-like environment. By successfully demonstrating the feasibility of utilizing soft robots for colon sampling, this work introduces a novel method for non-destructive inspection and sampling in the colon. It represents a significant advancement in the field of medical robotics, offering a potential solution for more efficient and accurate examination and diagnosis of intestinal diseases, specifically for colorectal cancer.

## 1 Introduction

The World Health Organization’s International Agency for Research on Cancer released global cancer statistics in 2020, revealing that China accounted for approximately 555,000 new cases of colorectal cancer, representing 12.2% of all new cancer cases. The incidence rate of colorectal cancer in China is increasing annually and poses a significant threat to public health. Timely colon examination and sampling are crucial steps in preventing, diagnosing, and treating colorectal cancer. Currently, colonoscopy-based examination and sampling are widely used and considered the “gold standard” for colorectal cancer screening (ASGE Technology Committee et al., 2015; Akyuz and Akyuz, 2016). However, colonoscopy can cause discomfort, mucosal tissue damage, and even intestinal perforation (Fan et al., 2023). Another method, capsule endoscopy, involves swallowing a capsule equipped with a camera to observe the intestines, but it has limitations such as the inability to focus on specific areas and the potential for missing lesion locations.

Fortunately, Soft robots offer a promising approach to intestinal disease examination and sampling due to their high flexibility and ability to safely interact with humans. Soft robots are defined as systems that utilize hyperelastic materials with a Young’s modulus range similar to that of natural soft biological tissues (10^4^–10^9^ Pa) as their body or primary components (Rus and Tolley, 2015; Lee et al., 2017). They possess infinite degrees of freedom (Bruder et al., 2019), enabling them to deform in various complex environments, including narrow areas and extreme temperatures (Tolley et al., 2014). Compared to colonoscopy and capsule endoscopy, soft robots have several advantages in colon examination and sampling. Firstly, their manufacturing costs are low due to the utilization of inexpensive source materials and uncomplicated manufacturing techniques, making them single-use and eliminating the need for frequent disinfection (Franchetti and Kress, 2017); Secondly, the soft robot’s body material has a Young’s modulus similar to human tissue, reducing the risk of intestinal tissue damage and minimizing patient discomfort; Finally, soft robots can be controlled and autonomously navigate the intestine, enabling targeted examination of diseased areas.

Many significant progresses have been made in the development of intestinal soft robots. Some studies have focused on designing soft robots for colonoscopy, demonstrating their ability to navigate in PVC pipes successfully (Alcaide et al., 2017). However, these studies still face challenges in replicating the real intestinal environment and achieving efficient performance. Recent research has explored the role of intestinal mucus and developed intestinal fluid simulations to evaluate the feasibility of soft robots operating in a realistic intestinal environment. Snail-like soft robots have been proposed and shown to operate autonomously in these simulations, offering a potential alternative to traditional gastrocolon examinations (Chan et al., 2018). Moreover, closed-loop control has been introduced to enhance the capabilities of intestinal soft robots. This control method allows the soft robot to adjust its deformation in real time based on the forces exerted on the intestinal wall. Inchworm-like soft robots have also been developed for colonoscopy, demonstrating smooth movement in variable-diameter pipes, as well as successful navigation through large bends (Manfredi et al., 2019). These soft robots effectively minimize pressure on the intestinal wall and reduce patient discomfort during examinations. While these studies have significantly advanced soft robot technology and intestinal disease examination and diagnosis, they primarily focus on movement and observation within the intestine or intestinal-like environments, lacking additional functions such as sample collection.

To address this gap, this paper proposes an colon sampling soft robot inspired by earthworm. The robot is capable of controlled movement and sample collection in an intestinal-like environment. It consists of a robot body with an earthworm-like structure for operation in narrow and soft pipe environments, and a sampling part with a flexible arm structure resembling an elephant trunk for bidirectional bending sampling. The feasibility of the soft robot’s controllable operation and sampling in an intestinal-like environment is validated through a combination of finite element simulation and experiments, providing a novel approach for soft robot functional in the intestine.

## 2 Design and production of the colon sampling soft robot

### 2.1 Design of the colon sampling soft robot

Nature has long provided inspiration for the design and fabrication of soft robots, with creatures like inchworms (Guo et al., 2017), octopuses (Cianchetti et al., 2015) and snakes (Yang et al., 2018), offering valuable insights. Given the objective of this study, which is to explore the use of soft robots for collecting samples in the intestinal environment, it is important to consider that the intestinal tract is a slender tubular structure, much like the narrow environment in which earthworms move within the soil. Therefore, mimicking the peristaltic motion of earthworms becomes a suitable choice for developing soft robots tailored for intestinal applications. Earthworms possess longitudinal and circular muscles in their body segments. When the longitudinal muscles contract and the circular muscles relax, the body segments of the earthworm shorten, increasing the pressure in the body cavity and extending the setae on the body surface outward to anchor themselves in the surrounding environment. Conversely, when the longitudinal muscles relax and the circular muscles contract, the body segments of the earthworm elongate, reducing the pressure in the body cavity, and causing the setae anchored to the surroundings to retract. By alternating the contraction of these two muscle types in adjacent body segment groups, earthworms achieve a wave-like forward movement.

**FIGURE 1:**
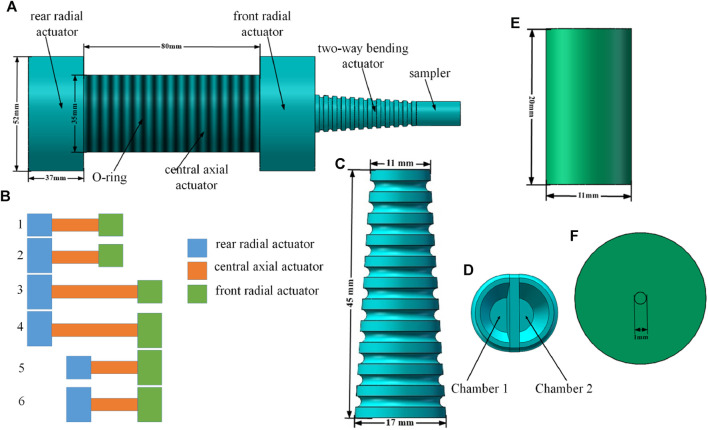
Structure and locomotion gait of the colon sampling soft robot. **(A)** Structure of the soft robot, **(B)** Motion cycle of body part of the soft robot, **(C)** Front view and **(D)** Sectional view of the bending actuator, **(E)** Vertical view **(F)** Front view of the sampler.

Taking inspiration from this earthworm-like movement mechanism (Calderón et al., 2016), the body of the colon sampling soft robot in this study is composed of three segments: a front radial actuator, a central axial actuator, and a rear radial actuator (as shown in Figure 1A). The radial actuator serves a function similar to the circular muscles of earthworm, while the axial actuator mimics the longitudinal muscles. The axial actuator is encased in a nitrile rubber O-ring, allowing it to primarily expand and contract in the axial direction during operation, with minimal radial deformation. As for the radial actuator, due to its small axial length and its bonding with the axial actuator at one end, it mainly expands in the radial direction upon activation, with negligible axial deformation.

To better depict the movement mechanism of the soft robot body, Figure 1B presents a comprehensive cycle of its motion. Step 1 of Figure 1B represents the initial state of the soft robot, while steps 2–6 in Figure 1B illustrate the five distinct states during movement. The specific description of each state is as follows:

Step 1 The initial state of the soft robot.

Step 2 The chamber of the rear radial actuator is inflated, securing it to the inner wall of the pipe.

Step 3 The central axial actuator expands, propelling the soft robot forward along the axial direction.

Step 4 The chamber of the front radial actuator is inflated, causing it to expand radially and anchor to the inner wall of the pipe.

Step 5 The chambers of the rear radial actuator and the central axial actuator are deflated, and the soft robot advances as a whole.

Step 6 The rear-end radial actuator expands radially and is anchored to the inner wall of the pipe.

The sampling part of the robot is fixed at the front end of the radial actuator in the body of the soft robot (as depicted in Figure 1A). The sampling part consists of a two-way bending actuator and a soft sampler. The two-way bending actuator utilizes a soft structure resembling an elephant trunk, as shown in Figure 1C. The outer part of the actuator takes the shape of a truncated cone, while the inner part is divided by an intermediate wall, forming two symmetrical sealed chambers on the left and right sides (as shown in Figure 1D). By introducing a certain amount of gas into one of the chambers, the two-way bending actuator bends towards the opposite side due to the pressure difference between the chambers. To achieve this, the outer wall and middle wall of the two-way bending actuator are constructed using Ecoflex 00-30 and Dragon Skin 10 silicone rubber, respectively. The difference in Young’s modulus between these two materials enhances the bending effect of the two-way bending actuator.

**FIGURE 2:**
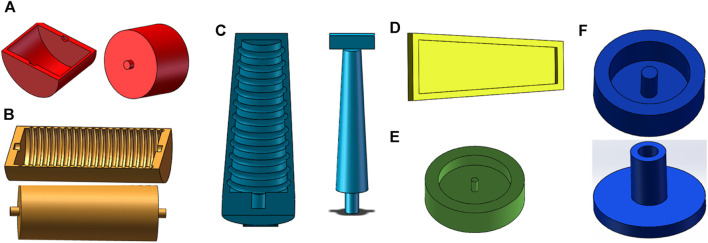
Molds for fabrication of the soft robot. Molds of the radial actuator **(A)**, axial actuator **(B)**, outer wall of the bending actuator **(C)**, inner wall of the bending actuator **(D)**, top layer of the sampler **(E)**, bottom layer of the sampler **(F)**.

For effective collection of intestinal samples, the soft robot’s sampler must not only carry out sample collection but also ensure the integrity of the collected samples until the robot exits the intestinal environment. Considering the viscous nature of intestinal fluid, aspiration is employed for sampling. The sampler is designed as depicted in Figures 1E, F, consisting of a top cover and a bottom barrel structure. When these two components are joined together, a sealed chamber is naturally formed inside, serving as a sampling chamber to store the collected samples. The top cover of the sampler is made of Ecoflex 00–30, while the remaining parts of the sampler utilize Dragon Skin 10. A small hole with a diameter of 1 mm is reserved at the center of the top cover of the sampler. When the top of the soft sampler is fully immersed in the intestinal mucus, the sampler is pumped, creating a pressure difference that allows the mucus to flow into the sampler chamber. Due to the small diameter of the reserved hole, the mucus entering the chamber is prevented from overflowing. Once the mucus collection is completed, the soft robot for colon sampling carries the sample and exits the intestinal environment, thereby concluding the process of collecting intestinal samples. Figure 2.

### 2.2 Production of the colon sampling soft robot

The production of an colon sampling soft robot is accomplished using casting molding technology. The specific steps involved in the production process are as follows:

1) Mold Design: SolidWorks software is utilized to create the production mold for the colon sampling soft robot. Figures 2A, B depict the models for the radial actuator and axial actuator, respectively. The molds serve the purpose of manufacturing the semi-cylindrical shell of the corresponding actuator. The complete actuator constitutes a cavity structure created by joining two of these shells together. Figures 2C, D represent the models for bidirectional bending. The actuator employs a layered manufacturing technique, where the curved part and the restricted part are manufactured independently. Figure 2C illustrates the mold used for producing the curved part, while Figure 2D showcases the mold used for manufacturing the restricted part. These models are used for the outer wall, middle wall, cover, and bottom layer of the sampler, as shown in Figures 2E, F. To ensure structural integrity, the wall thickness of the mold is set at 2 mm, which is more than three times the nozzle diameter of the 3D printer used in this design (0.4 mm) (Yap et al., 2016). And all molds can be used repeatedly.
2) Mold Manufacturing: The mold is manufactured using 3D printing technology with the HORI Z300 printer, purchased from Beijing Huitianwei Technology Co., Ltd. The key printer parameters are set as follows: a model layer height of 0.1 mm, a print head working temperature of 210°C, a printing platform operating temperature of 40°C, and a filling and printing speed of 100 mm/s.
3) Structural Pouring: Firstly, a suitable amount of white petroleum jelly release agent is applied to the surface of the prepared mold to facilitate demolding. Next, equal weights of component A and B materials are weighed and thoroughly stirred for 3 min using a stirring rod. The liquid silicone rubber material is then degassed in a vacuum degassing equipment for 10 min to eliminate any bubbles. Subsequently, the degassed silicone rubber is poured into the mold and heated at 60°C for 30 min to complete the curing process. Finally, the cured silicone rubber is removed from the mold.
4) Structural Assembly: The completed actuators are bonded together using Sil-Poxy silicone rubber adhesive, following the structure depicted in Figure 1A. Additionally, an O-ring is placed around the central axial actuator.

### 2.3 Construction of the control system

**FIGURE 3:**
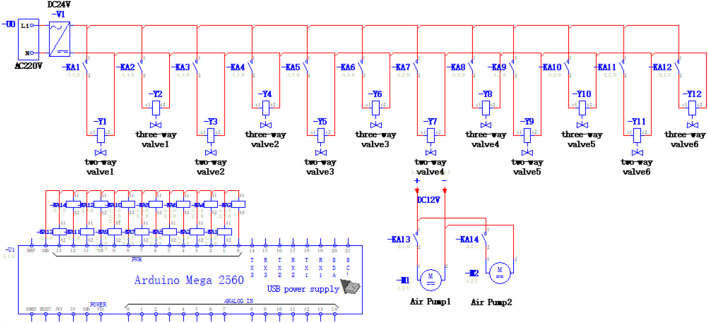
Electrical control schematic diagram of the soft robot.

The automatic navigation and sample collection of the soft robot are conducted through open-loop control. There are two primary methods employed for controlling the soft robot: i) Pre-determined operation: The purpose and trajectory of the soft robot’s work are predefined, allowing the soft robot to autonomously finish the entire operation process by setting the gait and duration of its movements. ii) Remote manipulation: The soft robot can also be remotely controlled in real-time for its movements and works. The control system of the soft robot comprises a control unit and a drive unit. The control unit consists of a host computer, a microcontroller, and a relay, while the drive unit includes an air pump, a throttle valve, and a solenoid valve, as shown in Figure 3. The control principle of this system is as follows: the relay receives signals from the compiled program in the microcontroller, enabling logical control of the solenoid valve and air pump in a sequential manner. This control mechanism facilitates functions such as air intake, air release, and status maintenance of the actuator. The actuator’s movement sequence aligns with the driving sequence, ensuring the soft robot moves forward until it reaches the target position. If the driving sequence is reversed, the soft robot will move backward until the situation is clear.

The solenoid valve group in the control system comprises a two-position two-way solenoid valve and a two-position three-way solenoid valve. These valves enable the three required states for the operation of the colon sampling soft robot: chamber expansion, chamber contraction, and chamber status maintenance. When both the two-way valve and the three-way valve are energized, gas flows into the actuator’s chamber. When the two-way valve is de-energized and the three-way valve is energized, the gas is retained in the chamber. Conversely, when the two-way valve is energized and the three-way valve is de-energized, the remaining gas in the chamber is discharged through the three-way valve. The throttle valve regulates gas flow in the air circuit, preventing damage to the actuator caused by excessive pressure or insufficient expansion. The input end of the two-way valve is directly connected to the output end of the throttle valve, and the air output from the two-way valve flows into the three-way valve. Subsequently, the gas enters each chamber of the colon sampling soft robot.

## 3 Numerical simulation of the colon sampling soft robot

**FIGURE 4:**
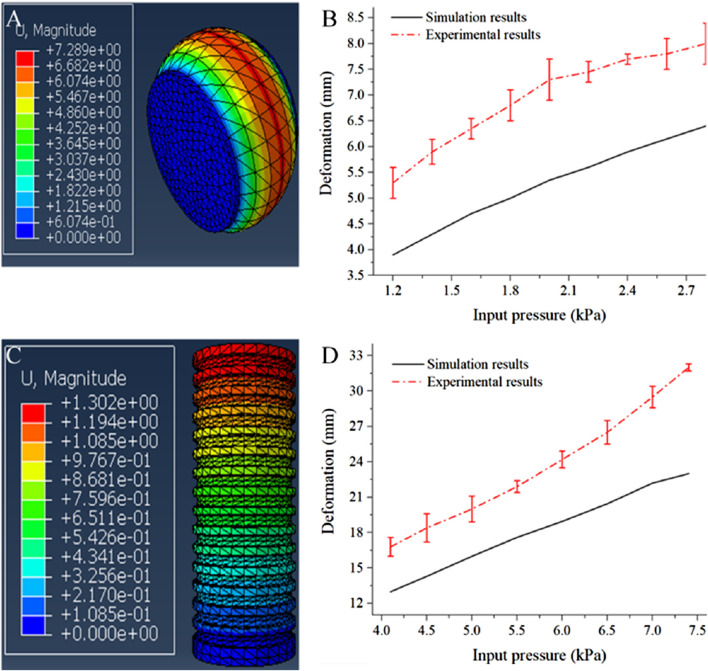
Numerical simulation results of the radial actuator and axial actuator. **(A)** Numerical simulation result of the radial actuator, **(B)** Deformations of the radial actuator under different input pressures, **(C)** Numerical simulation result of the axial actuator, **(D)** Deformations of the axial actuator under different input pressures.

The deformation performance of each actuator in the colon sampling soft robot was simulated using finite element analysis software to verify the structural design and material selection. Abaqus software was used for the finite element analysis, and the Yeoh model with the parameters c_10_ = 0.01 and c_20_ = 0.02 (Yan et al., 2016) was employed as the strain energy density function model for Ecoflex 00–30. Dragon Skin 10 had a Young’s modulus of 201 kPa and a Poisson’s ratio of 0.49 (Veiga et al., 2021). The deformation performance of the radial actuator was simulated firstly. The simulation results, as shown in Figure 4A, indicate that the radial actuator expanded only in the radial direction and had minimal deformation in the axial direction, which aligned with the explanation in Section 2.1. And the relationship between the input air pressure in the radial actuator cavity and the deformations of the actuator was further investigated. Different air pressures were applied into the actuator and the corresponding deformations were measured, as shown in Figure 4B. The results show that the deformation has a linear relationship with the input air pressure into the actuator, and also displays relatively consistent with that of by actual experiments, because the error between the relative deformation acquired by numerical simulation and the relative deformation acquired by experiments is less than 3%.

Next, the axial actuator was simulated using the same material as the radial actuator. The simulation results of the axial actuator, displayed in Figure 4C, revealed that the actuator elongated solely in the axial direction and exhibited minimal radial deformation after pressurization. This aligns with the discussion in Section 2.1, confirming the intended deformation effect. Figure 4D portrayed the relationship between the input air pressure in the axial actuator cavity and its’ deformation, obtained through both numerical simulation and actual measurements. A comparison between the two lines indicated relatively consistent results due to the small relative deformation error.

**FIGURE 5:**
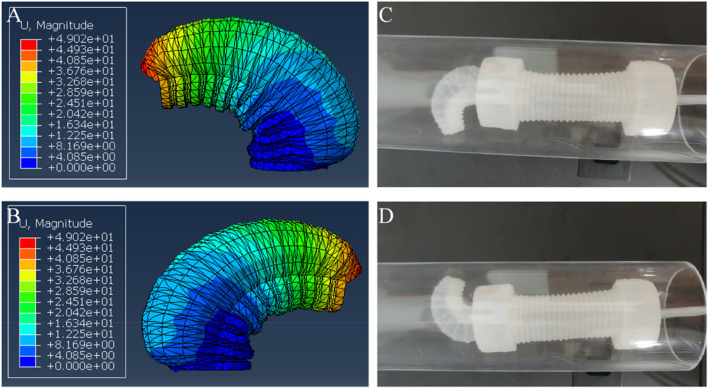
The bending performance of the bending actuator. **(A)** Numerical simulation result when the right chamber was applied with air pressure, **(B)** Numerical simulation result when the left chamber was applied with air pressure, **(C)** Experimental result when the right chamber was applied with air pressure, **(D)** Experimental result when the left chamber was applied with air pressure.

The numerical simulation results of the two-way bending actuator, depicted in Figures 5A, B, illustrated the actuator’s bending behavior when air pressure was input into the right and left chambers. The actuator exhibited a bending angle of approximately 90°, and the magnitudes of the two deformations were completely consistent, effectively verifying the structure’s bidirectional bending capability. Moreover, the actual bending performance of the two-way bending actuator was experimentally tested, as shown in Figures 5C, D. The bending effect observed through numerical simulation closely matched the actual measurement, further confirming the design’s rationality.

## 4 Experimental results

### 4.1 Motion experiments in rigid pipe

**FIGURE 6:**
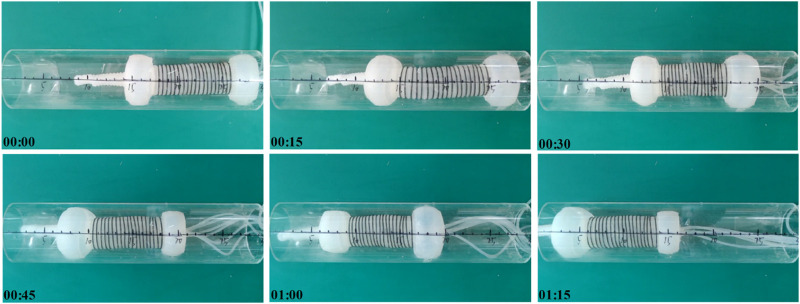
Moving experiment of the soft robot in a rigid pipe.

The objective of this experiment is to demonstrate the controlled movement capability of the colon sampling soft robot in a rigid pipeline. Although there are distinctions between the pipeline environment and the intestinal environment, this experiment serves to verify the functionality of the drive system in the designed colon sampling soft robot. Figure 6 displays the state diagram of the soft robot at specific moments during the experiment with a time interval of 15 s between each picture. Based on calculations, the movement speed of the colon sampling soft robot in the rigid pipe is approximately 11.2 ± 1.1 cm/min. This experiment provides preliminary evidence that the colon sampling soft robot can execute controlled movements in a rigid pipeline.

### 4.2 Motion experiments in flexible pipe

**FIGURE 7:**
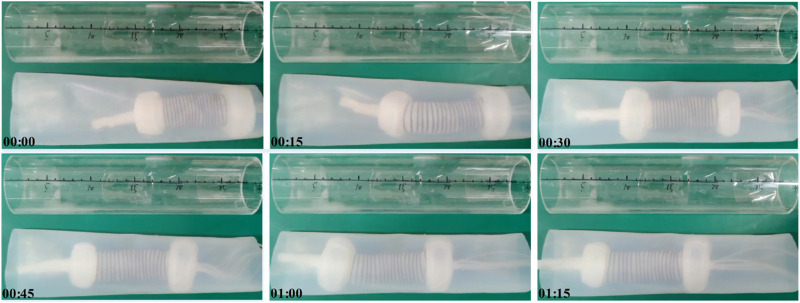
Moving experiment of the soft robot in a flexible pipe.

Given the inherent softness of the intestine, the performance of the colon sampling soft robot was further evaluated in a silicone hose with an inner diameter of 65 mm. Figure 7 illustrates the movement process of the soft robot in the flexible pipe, with a time interval of 15 s between each picture. The calculated movement speed of the colon sampling soft robot in the silicone hose is approximately 9.0 ± 0.7 cm/min. The results indicate that the movement speed of the soft robot in the flexible pipe is lower than that in the rigid pipe. This disparity is due to the corresponding deformation of the flexible pipe during the actuator’s expansion and contraction movements. Nevertheless, the experiment confirms that the colon sampling soft robot possesses the capability to perform controlled movements in flexible tubes. In contrast to the rigid pipe environment, the flexible pipe necessitates the radial actuator of the robot to allocate more time for anchoring, consequently leading to a reduced relative movement speed of the soft machine within the flexible pipeline.

**FIGURE 8:**
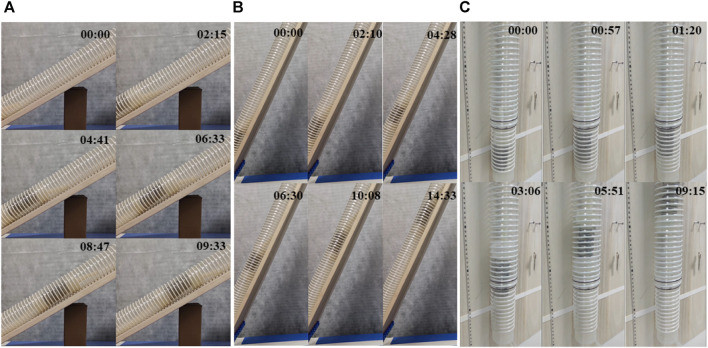
Moving experiment of the soft robot in a flexible pipe that had a 30-degree slope **(A)**, 60-degree slope **(B)** and variable diameter **(C)**, respectively.

Moreover, the inner surface of the intestine exhibits slopes at various locations rather than being entirely flat and the intestinal diameter is also constantly changing. Consequently, to further investigate the adaptability of soft robots within the intestine, we conducted tests to assess the operational efficacy of a soft robot in a flexible pipe positioned at an angle and within variable diameter flexible pipes, as illustrated in Figure 8. Figures 8A, B depict the crawling performance within a pipe inclined at 30 and 60°, respectively. Figure 8C shows the effect of crawling in a flexible pipe with varying diameters. These results indicate that the soft robot can navigate in flexible pipes within a specific range of angles and variable diameter, achieving crawling speeds of 2.95 ± 0.7 cm/min and 3.03 ± 0.4 cm/min in the respective inclined flexible pipes and 3.24 ± 0.2 cm/min in the flexible pipe with the variable diameters.

### 4.3 Experiments on collecting mucus samples

To further explore the sampling capabilities of soft robots, this experiment involved driving the soft robot to collect intestinal fluid simulants in an environment resembling the intestine. Initially, a flexible pipe with an inner diameter of approximately 60 mm was created using plastic film. Folds were intentionally introduced to enhance its suitability for the intestinal environment. Prior to the commencement of the experiment, a 100 mL sample of a 3% concentration magnesium lithium silicate solution, simulating human intestinal fluid (Xin et al., 2020), was introduced into the pipe.

**FIGURE 9:**
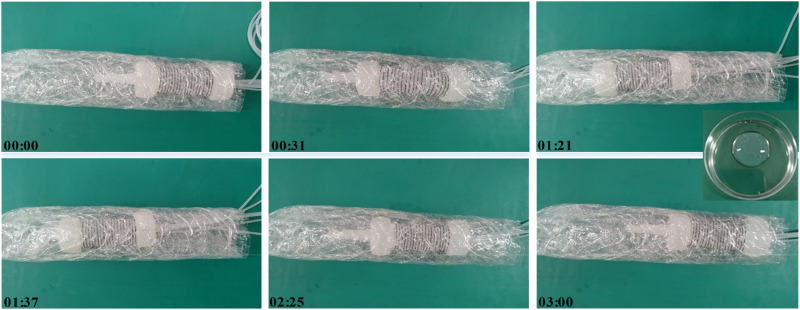
Sampling experiment of the soft robot.

Figure 9 depicts the state diagram of the colon sampling soft robot at six moments during the sampling experiment, including the soft robot’s entry into the pipeline, sample collection, and exit from the pipeline. The soft sampler functions as an autonomous chamber featuring a small aperture at one end. And its front end connects to the end of the bending actuator, and it also consists of a trachea that connects the sampler’s chamber. The front end of the trachea operates with an independently controlled pneumatic system. During the sampling procedure, the sampler’s end is positioned at the sampling location, and subsequently, the pneumatic system generates negative pressure within the sampler chamber, facilitating the suction of the sample into the sampler. To extract the sample, applying positive pressure within the chamber is sufficient to expel it from the sampler. Notably, the soft robot collects samples at the 3rd and 4th moments, acquiring samples from the right and left sides of its body.

Following the soft robot’s exit from the pipeline environment, the sample within the sampling chamber is expelled, as shown in the inset in Figure 9. This experiment validates the successful completion of sample collection in an environment resembling the intestine using the designed colon sampling soft robot. The sample collection further confirms the feasibility of the soft robot for colon sampling.

## 5 Conclusion

To meet the requirements of intestinal disease examination and sampling, this study presents the design of an earthworm-like soft robot for colon sampling, employing gas drive mode and casting molding process. Finite element analysis is employed to simulate the performance of each actuator of the soft robot, thereby validating the design’s rationality. The soft robot demonstrates controlled movement capabilities within both rigid and flexible pipes, achieving speeds of 11.2 cm/min and 9.0 cm/min, respectively. Additionally, through the mucus sampling experiment using plastic film pipes, the feasibility of applying the colon sampling soft robot to sample collection within an environment resembling the intestine is effectively established. This work represents a step toward the novel solution to the non-destructive inspection and sampling of the intestine, and a new intestinal soft robot with sampling functionality.

## Data Availability

The raw data supporting the conclusions of this article will be made available by the authors, without undue reservation.
